# Vigour in active avoidance

**DOI:** 10.1038/s41598-017-00127-6

**Published:** 2017-03-03

**Authors:** Camilla L Nord, Gita Prabhu, Tobias Nolte, Peter Fonagy, Ray Dolan, Michael Moutoussis

**Affiliations:** 1 Institute of Cognitive Neuroscience, University College London, London, UK; 2 Wellcome Trust Centre for Neuroimaging, UCL, 12 Queen Square, London, UK; 3 Anna Freud Centre, London, UK; 4 Research Department of Clinical, Educational, and Health Psychology, University College London, London, UK; 5 Max Plank UCL Centre for Computational Psychiatry and Ageing Research, London, UK

## Abstract

It would be maladaptive to learn about catastrophes by trial and error alone. Investment in planning and effort are necessary. Devoting too many resources to averting disaster, however, can impair quality of life, as in anxiety and paranoia. Here, we developed a novel task to explore how people adjust effort expenditure (vigor) so as to avoid negative consequences. Our novel paradigm is immersive, enabling us to measure vigor in the context of (simulated) disaster. We found that participants (N = 118) exerted effort to avoid disaster-associated states, adjusting their effort expenditure according to the baseline probability of catastrophe, in agreement with theoretical predictions. Furthermore, negative subjective emotional states were associated both with threat level and with increasing vigor in the face of disaster. We describe for the first time effort expenditure in the context of irreversible losses, with important implications for disorders marked by excessive avoidance.

## Introduction

The psychobiological mechanisms serving to avoid adversity are under-investigated in humans, mainly due to it being unethical to expose people to potential harm in order to study the optimal vs. suboptimal active avoidance of highly salient aversive events (disasters). However, this topic is of great importance, as key symptoms common across several neuropsychiatric conditions involve a counterproductive anticipation of disaster. Examples include obsessive thoughts, paranoid beliefs, and anxious catastrophizing. In obsessive-compulsive disorder (OCD), patients are often consumed with fears of harming others, and develop maladaptive avoidance behaviour which they believe will reduce the probability of harming other people^1–3^. Patients with psychotic depression commonly experience nihilistic-type delusions^4^ about impending disaster, or delusions about guilt^5^. In extreme scenarios, both disorders can result in patients remaining housebound because of their repetitive thoughts about possible injuries they may inflict on others^3^. Addressing these symptoms experimentally requires developing ecologically valid cognitive paradigms that evoke anticipation of harm to others, and measuring adaptive defensive behaviours. In doing so, we can also quantify avoidance behavior in healthy humans and progress towards assessing its role in transdiagnostic symptom dimensions^6, 7^.

## Vigor and reward

Most decision-making studies in humans focus on *which* of a number of actions to make. With respect to many goals, particularly keeping safe, the dimension of how vigorously to act is equally as important. In the context of disaster, the preferable choice is self-evident, but the value of the choice with respect to vigor, or effort, is more complex: reinforcement learning literature indicates that computing vigor involves balancing two costs: the opportunity cost of slow responding, versus the energy costs associated with vigorous responding. These ideas echo earlier theoretical and empirical work on motivation, which argues that individuals follow an effort conservation principle, investing only the minimum effort required for successful goal attainment^8, 9^.

Reinforcement learning models of vigor provide a computational framework explaining how individuals might balance these costs and benefits of quick responding^10^. The models assume that subjects maximize the average rate of net utility per unit time. In the case of threat, the opportunity and performance costs of defensive activity must be balanced against the considerable, albeit infrequent, cost of disastrous outcomes^11^. Higher cost is associated with more vigorous, frequent actions.

Previous work has provided evidence in support of this framework in the domain of opportunity. In one study, participants adjusted their mean response times according to the local average reward rate^12^. Thus, when participants had earned more money in the preceding few minutes, they exerted more effort in the form of faster responses where the experimental design was such that this did not produce more reward, consistent with the animal literature. For example, animals will work harder when they are hungry, even for a reward like water which does not alleviate hunger^13^. The energizing of responding by reward thus has a Pavlovian component, rather than being purely instrumental. This can be thought of as an effective prior belief that the right thing to do in a rewarding context is to increase motor vigour.

Most previous empirical studies have explored vigor specifically in the context of reward^12, 14–17^, despite the prominent role of avoidance behaviour in some neuropsychiatric disorders. Further, characterization of subjective states has also been coarse or absent. Perhaps most crucially, human studies typically use small monetary incentives, which have low ecological validity for catastrophic events. There is evidence that monetary rewards and punishments do not evoke the same neural responses as primary reinforcers (e.g., food)^18^. Most persuasively, work on motivational intensity found that using elating materials as a reward reversed motivational deficits normally seen in dysphoric individuals^19^. However, it is unclear how emotionally negative outcomes motivate behavior. There is a methodological need to develop paradigms that characterize effortful behavior in the context of realistic, non-monetary outcomes.

To address these gaps we developed a novel, immersive, paradigm designed to measure vigor while evoking a context of irreversible loss. We measured the effort (grip force) subjects exerted to prevent a (virtual) dog being run over by a car, while manipulating the probability of the occurrence of this negative event. We acknowledge that participants were most likely aware of the demand characteristics of the task. However, this was in keeping with our effort to model true events, as demand effects would contribute to an irreversible cost in a real situation (for example, if the experimenter’s dog was in danger of being run over). The task followed a written exercise and personalized selection of stimuli to maximize immersiveness, as described below. We aimed to establish a realistic environment to measure whether or not participants adjust effort according to the baseline probability of experiencing an aversive outcome, as monetary reward paradigms and computational models predict. To our knowledge, this is the first investigation of vigor in the context of highly salient, aversive events (‘disasters’).

Our paradigm differs in several important ways from those employed previously: first, our use of non-monetary salient outcomes, to better evoke brain states associated with true disaster anticipation; second, we use these outcomes to study vigour; and lastly, our immersive design of the task uses concurrent emotion measures. These factors enable us to test predictions about instrumental aversive vigour and its relation to subjective emotions.

We hypothesized that participants would exert greater effort in the higher-disaster-probability context, thus tending to optimize average outcomes. We also hypothesized that effort exerted to avoid disaster would strongly relate to subjective emotionality. We measured several negative emotions (fear, anger, etc.) throughout the task and predicted the strength and valence of emotion (whichever specific words used to express it), to correlate with behavioural motivation (effort). Thus, we could measure whether the participants who were most emotionally affected by ‘disasters’ were also used the most physical effort to avoid aversive outcomes.

## Method

### Participants

One hundred and eighteen participants were recruited from the subject pool associated with University College London’s Psychology Department. We ran two iterations of the experiment, which differed in: (1) sample size and (2) number of trials. Experiment 1 was run on twenty-eight subjects (mean age = 27.57, *SD* = 12.09; 24 female), utilizing 400 trials. After verifying that our effect could be found in fewer trials, Experiment 2 was run on 90 new participants (mean age = 25.11, *SD* = 6.30; 67 female), using a 120-trial design. Participants provided written informed consent and were compensated for their time. Both experiments were conducted in accordance with the University College London ethics guidelines: Experiment 1 was approved by the London Queen Square Ethics Committee (Ethics No. 3450/002) and Experiment 2 by the University College London Graduate School Ethics Committee (Ethics No. 6129/002).

#### Sample size

We ran two power calculations to determine the sample sizes of Experiment 1 and 2, using G*Power 3.1.9.2; statistical test: difference between two dependent means (matched pairs). Experiment 1 was powered to detect a moderately-sized within-subjects effect: to detect a Cohen’s d of 0.6 with 80% power, we would need N = 24 (we used N = 28 to allow for attrition). While this sample size was sufficient to detect our primary effect, it was underpowered to detect correlation effects which would allow us to explore the relationship between behavior and subjective emotion. We therefore calculated that for a smaller effect size, Cohen’s *d* = 0.3, in a correlation test and 80% power, we would need 82 participants. For this reason, we recruited 90 new participants for Experiment 2 (mean age = 25.11, *SD* = 6.30; 67 female) with the aim of replicating our original effect and testing these subtler relationships.

### Procedure

All participants first performed a practice monetary version of the paradigm. This was followed by immersive procedures to induce a state primed for averting catastrophe and maximizing sensitivity: (1) completing a written immersion exercise; (2) selecting idiographic emotional words. These were incorporated into in-task emotion questions in 15% of trials; and (3) choosing their favorite dog, from a selection of four photographs, which would be their primary stimulus in the task. Participants then started the main task, which took place in blocks with short breaks in the middle.

#### Grip force calibration

To calibrate the grip force to each participant’s strength, participants were instructed to grip with their maximum strength for a six-second period. This process (a screen reading “get ready to squeeze” followed by “squeeze as hard as you can!!!”) was repeated three times. The maximum force achieved out of the three trials was used to calibrate the force necessary to “save the dog” in the main experiment.

#### Monetary practice task

Before starting the main task, the experiment was explained in detail and all participants completed a 15-trial version with monetary stimuli to ensure understanding of the trial structure and squeeze grip manipulation. In this practice version of the task participants had to prevent a computerized ‘thief’ from stealing a bag of gold by squeezing the grip to ‘sound an alarm’. Each participant experienced both a high-risk and low-risk condition where the money was more or less likely to get stolen, respectively, if the subject squeezed below threshold. This mirrored the statistics of our main task. We confirmed orally that subjects fully understood the structure of the experiment and re-ran the practice task if a participant did not fully understand.

#### Personalization and immersion

**Figure 1:**
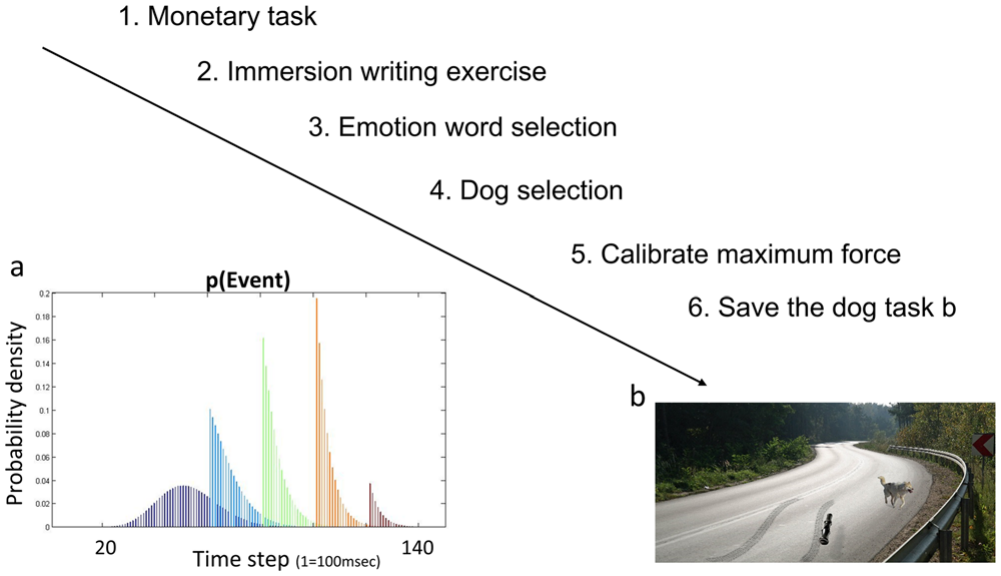
Task stages. Participants first completed a practice monetary task, then an immersion exercise writing about a pet (real or hypothetical) who died, then briefly selected preferred emotional words (for use in the in-task questions), then selected a preferred dog, and finally exerted their maximum force before beginning the task (1b). How the conditional distribution of the car arriving changed with time within a trial is shown in 1a. Each trial was structured in 100 msec time steps. The distribution at 0 sec is shown in purple; this gradually changes, given that no car arrives, to the brown at *t* = 12 sec. (based on previous work^11^). For most trials, the more time had passed, the more likely it was that the car was about to arrive (blue to orange). Towards the very end of the trial, however, it became more likely that no car would appear at all. The following image used in Fig. 1b is reproduced under the terms of a Creative Commons 2.0 generic license (https://creativecommons.org/licenses/by/2.0/legalcode) and has been adapted from its original form (Original Author: Drew Avery; title: Annual Dog Sled Race). The link to the original image is: https://www.flickr.com/photos/33590535@N06/5391571785/in/photostream/ The following image used in Fig. 1b is reproduced under the terms of a Creative Commons Attribution-Share Alike 3.0 Unported license (https://creativecommons.org/licenses/by-sa/3.0/legalcode) and has been adapted from its original form (Original Author: Sebastian Hartlaub; title: Dog ultrasound whistle). The link to the original image is: https://commons.wikimedia.org/wiki/File:Hundepfeife05.JPG. The following image was reproduced under the terms of a Freeimages Content License (Author: http://www.freeimages.com/photographer/jakubson-56068). The link to the original image is: http://www.freeimages.com/photo/double-curves-1448529.

The main experimental task had several preparatory components (see Fig. 1): (1) a written immersion exercise; (2) selection of favorite dog for use in the task; and (3) personalization of in-task emotion questions. The purpose of these was to increase the immersive experience of the task, and better imitate highly salient negative events in the real world. Following these three components, participants completed the full task, which consisted of four blocks with intermittent emotion questions.

Participants were introduced to the task concept using the following exercise: “For the next minute, please think about the following scenario: you or someone you are close to has a long-time pet, who sadly passes away. Please write a couple phrases about how you would feel about this event, and how you would have felt if you could have prevented this event.”

See Supplementary Materials S1 for example responses.

Following this written task, participants were instructed to select their preferred dog out of four possible photos. Lastly, participants answered five questions about the initial exercise, which were phrased as “Which word describes best how you felt during the task?” Participants selected one word from each of five categories of emotion words derived from the Positive and Negative Affect Schedule (PANAS^20^, which comprised all four ‘Basic Negative Emotion Scales’ (Fear, Hostility, Guilt, and Sadness), and one ‘Basic Positive Emotion Scale’ (Joviality). Each category had between 5 and 8 possible words to choose from. Each participant later answered in-task questions phrased with the vocabulary he or she had selected (see Supplementary Materials S2 for full word list).

#### Main experimental task

**Figure 2:**
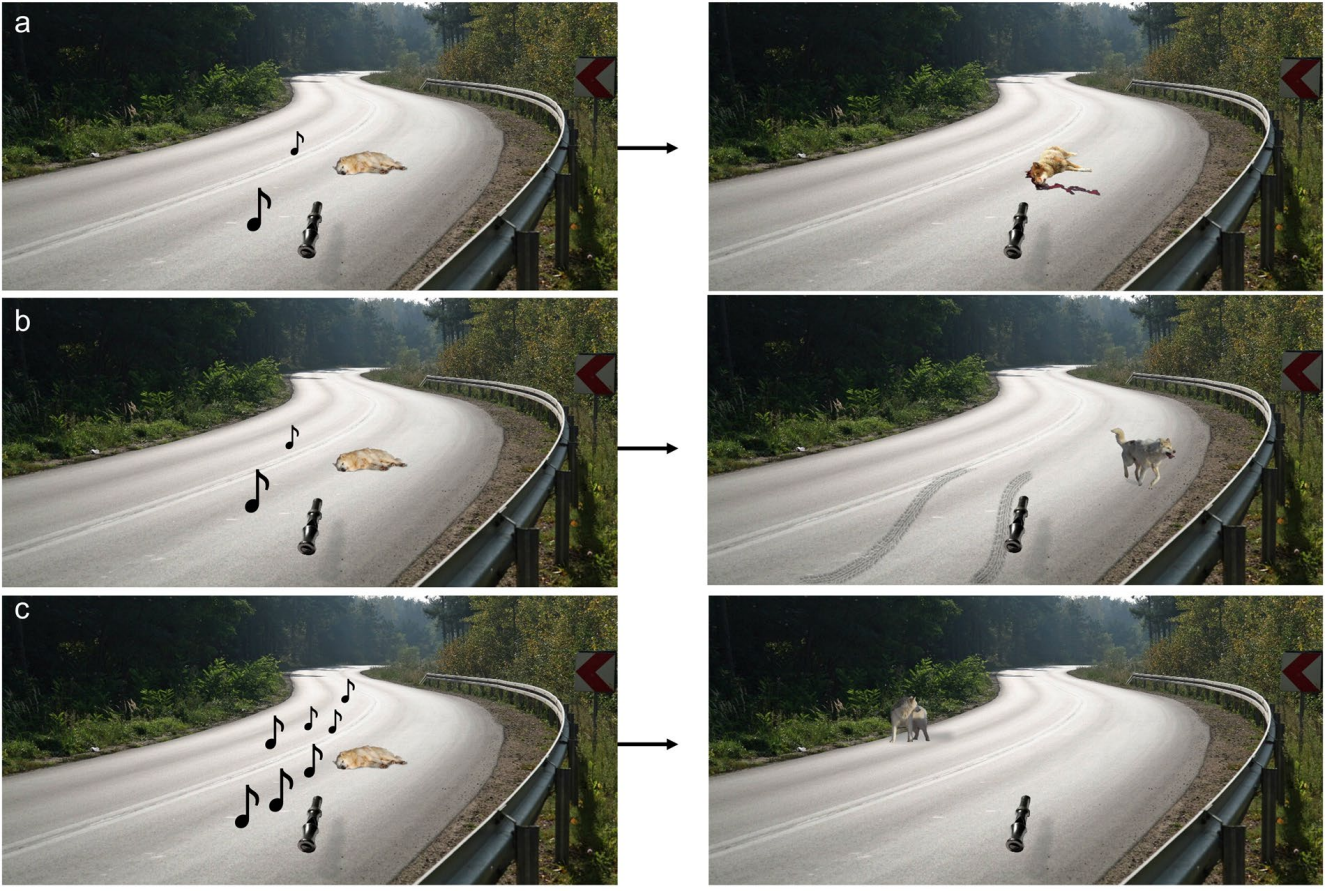
Example trials of main task. Each trial started with a view of the selected dog sleeping in the road; participants could squeeze a grip, producing a number of musical notes on the screen proportional to the strength of the squeeze grip (representing the strength of a dog whistle to wake the dog). (**a**) If insufficient force was exerted, the dog was killed by an oncoming car in 80% of high danger trials and 20% of low danger trials. (**b**) If insufficient force was exerted, the car swerved and the dog survived in 20% of high danger trials and 80% of low danger trials. (**c)** If the participant exerted a sustained force (calibrated to their individual grip strength), the trial resulted in the dog being saved. Less effort resulted in proportionally smaller probability of saving the dog. The car was assumed to come from behind the dog on the same side of the road (i.e., originating from beyond the bend in the lower left hand corner of Fig. 2a–c). All participants were UK residents, and were therefore familiar with cars driving on the left side of the road. The following images are reproduced under the terms of a Creative Commons 2.0 generic license (https://creativecommons.org/licenses/by/2.0/legalcode) and have been adapted from its original form (Original Author: Drew Avery; title: Annual Dog Sled Race). The links to the original images are: https://www.flickr.com/photos/33590535@N06/5391571793/in/photostream/ https://www.flickr.com/photos/33590535@N06/5391571785/in/photostream/ https://www.flickr.com/photos/33590535@N06/5392045246/in/photostream/. The following image is reproduced under the terms of a Creative Commons Attribution-Share Alike 3.0 Unported license (https://creativecommons.org/licenses/by-sa/3.0/legalcode) and has been adapted from its original form (Original Author Kim Hansen; title: Recently shot Greenland dog upernavik). The link to the original image is: https://commons.wikimedia.org/wiki/File:Recently_shot_Greenland_dog_upernavik_2007-07-02_edited.jpg. The following image is reproduced under the terms of a Creative Commons Attribution-Share Alike 3.0 Unported license (https://creativecommons.org/licenses/by-sa/3.0/legalcode) and has been adapted from its original form (Original Author: Sebastian Hartlaub; title: Dog ultrasound whistle). The link to the original image is: https://commons.wikimedia.org/wiki/File:Hundepfeife05.JPG. The following image was reproduced under the terms of a Freeimages Content License (Author: http://www.freeimages.com/photographer/jakubson-56068). The link to the original image is: http://www.freeimages.com/photo/double-curves-1448529. The musical note is a Unicode Character, ‘Eighth Note’ (U + 266 A) (The Unicode Standard, Version 1.1.0, (Mountain View, CA: The Unicode Consortium, 1993).

The main task consisted of a lifelike background of a road through the woods, where a dog was lying asleep. In each trial, participants were instructed to squeeze as hard as possible to activate a dog whistle in order to ‘wake the dog’, thus preventing it being run over by an oncoming car. The effort exerted was reflected by the number of musical notes that appeared on the screen, with a forceful enough squeeze representing the ‘loudness’ needed to ‘wake the dog’ (Fig. 2). Force was calibrated to each participants’ maximum grip strength (Fig. 2c). Pilot work indicated that this level of effort was too high to sustain throughout the experiment, which was confirmed by the finding in both experiments that participants performed well below ceiling (see Table 1). If the participant failed to squeeze hard enough, the outcome of the trial was displayed as a photograph: either the dog killed in the road, or swerving tire marks around a scared-looking dog (see Fig. 2).

**Table 1:** Full statistics for Experiments 1 and 2 for analyses of the effect of disaster condition on effort exerted.

	Normality	Effect of block number	Effect of disaster condition	Block-by-disaster interaction	Effect of age on disaster	Effect of gender on disaster
*Exp. 1*	D(28) = 0.113, *p* = 0.20	*F*(1,27) = 0.05, *p* > 0.250	*F*(1,27) = 10.33, *p* = 0.003	*F*(1,27) = 0.01, *p* > 0.250	*F*(1,14) = 0.704, *p* > 0.250	*F*(1,26) = 1.57, *p* = 0.221
*Exp. 2*	D(90) = 0.082, *p* = 0.177	*F*(1,89) = 0.17, *p* > 0.250	*F*(1,89) = 29.51, *p* < 0.001	*F*(1,89) = 0.08, *p* > 0.250	*F*(1,18) = 1.123, *p* > 0.250	*F*(1,88) = 0.96, *p* > 0.250

The probability of these two outcomes (swerve or kill) was explicitly given to each participant. In the two high-probability disaster blocks of 100 trials each (Experiment 1) or 30 trials each (Experiment 2), if the participant did not squeeze enough, the trial resulted in the ‘disaster’ outcome (the dog lying dead in the road) 80% of the time (Fig. 2a), while 20% of the time, the car “swerved” and the dog survived (Fig. 2b). These probabilities were reversed in the two low disaster probability blocks. At the beginning of each block, participants were told if the following block would be high- or low-traffic, and given the relevant probabilistic information. Trial outcomes (save/swerve/killed) took place within 25 seconds but did not affect the timing of subsequent trials. Force exerted (effort) was recorded during each trial in 100 ms bins. In between blocks 2 and 3, after participants had completed one high-traffic and one low-traffic block (i.e., halfway through the experiment), participants were given a rest of approximately ten minutes to recover from the physical exertion of the task, minimizing possible confounding by fatigue.

We based the structure of each trial on a normative theoretical model of vigor to avoid punishments^11^. At the beginning of each trial the key event (i.e., dog being killed or car swerving) had a bell-shaped expectation totaling just short of 100% (see Fig. 1a). Therefore the probability of a car appearing, given it had not yet appeared, initially increased with time. Towards the end of each trial, however, if a car had not yet appeared, the probability of it ever appearing decreased. For each participant the probability that a given effort level per timestep would save the dog remained constant between trials.

#### In-task emotion measures

Participants were presented with six emotion questions on 30% of the trials in the second half of each block. Questions were personalized using the individual participant’s earlier selections, so that, for instance, one person might be asked ‘To what extent did you feel afraid when trying to save the dog?’, while another might receive ‘To what extent did you feel scared when trying to save the dog?’, depending on the participant’s selection of ‘fear’ word from the Positive And Negative Affect Scale. In addition to the five individually-selected emotion words, all participants were also asked ‘To what degree did you feel in control when trying to save the dog?’.

### Materials

The task was coded in MATLAB (MATLAB and Statistics Toolbox Release 2012b, The MathWorks, Inc., Natick, Massachusetts, United States). This experiment was realized using Cogent Graphics developed by John Romaya at the Laboratory of Neurobiology at the Wellcome Department of Imaging Neuroscience. The squeeze grip used was custom-made at University College London (Institute of Neurology, 33 Queen Square, London, UK), and a National Instruments data acquisition device (DAQ) connected via USB port was used in conjunction with the MATLAB data acquisition toolbox.

### Data analysis

We calculated the average grip force exerted by each participant in each block, allowing us to compare vigor exerted during high- and low-probability disaster blocks. We also computed the average subjective emotion scores measured in-task to analyze how this variable was modulated by baseline disaster probability. Lastly, we examined the relationship between these two factors: whether the degree to which a subject altered his or her effort related to levels of subjective emotion.

Data were analyzed using MATLAB (2015) and the Statistical Package for the Social Sciences (IBM SPSS Statistics 22).

## Results

### Participants adjust effort according to baseline probability of ‘disaster’

#### Experiment 1

We analyzed participants’ average effort across the four blocks while correcting for each participant’s individually-measured maximum grip force. To do this, we computed average effort and normalized each participant’s mean effort by their respective in-task maximum effort. Thus, effort in each block was expressed as a ratio of effort-to-maximum effort; for example, 0.4 indicated a participant exerted on average 40% of their maximum effort capability.

**Figure 3:**
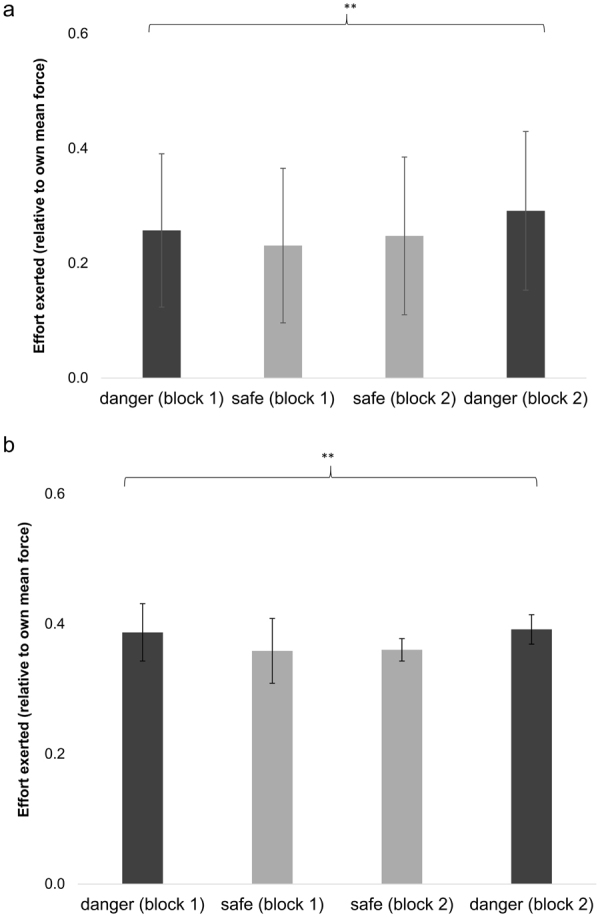
Effect of danger condition on force (expressed as proportion of subject’s maximum force). (**a**) In Experiment 1, there was a significant main effect of danger condition on force exerted. Participants exerted significantly more force during both high-danger blocks. (**b**) In Experiment 2, there was a similar main effect of danger, with participants exerting significantly more force during both high-danger blocks. *p = 0.003; **p < 0.001. Error bars represent 95% confidence intervals.

Full statistics are reported in Table 1, and outcomes experienced are presented in Table 2. We used a Kolmogorov-Smirnov test with Lilliefors correction to test normality of the main dependent variable, effort, indicating consistency with a normal distribution. We ran a 2-by-2 analysis of variance (ANOVA), which revealed no effect of block number (i.e., the first or second run of each condition) on effort. There was a substantial effect of the condition of interest, namely probability of disaster (*p* = 0.003, η_p_^2^ = 0.277): blocks with a higher probability of disaster evoked greater effort than those with a low probability of disaster. There was no interaction between block number and disaster condition, and no effect of age or gender on the main effect of disaster condition. A planned linear contrast revealed that participants exerted significantly more effort in blocks with a higher probability of disaster, *t*(27) = 3.21, *p* = 0.003. Eighty-six per cent of participants exhibited this trend (see Fig. 3a for overall performance). See Supplementary Materials S3 for how effort depended on time for a representative participant.

**Table 2:** Outcomes experienced in each condition (expressed as proportion of total outcomes).

	Outcome	Danger condition (95% C.I.)	Safe condition (95% C.I.)
*Experiment 1*	Save	0.65 (0.58–0.71)	0.57 (0.50–0.64)
	Swerve	0.06 (0.05–0.08)	0.35 (0.29–0.40)
	Killed	0.29 (0.24–0.34)	0.08 (0.07–0.10)
*Experiment 2*	Save	0.71 (0.62–0.81)	0.65 (0.54–0.75)
	Swerve	0.06(0.04–0.08)	0.29 (0.20–0.38)
	Killed	0.22 (0.15–0.29)	0.07 (0.43–0.09)

C.I.: 95% confidence interval. “Save” refers to the per cent of dogs actively saved by the participant in each condition; this only occurred when the participant exerted sufficient force. Otherwise, the car might swerve (more likely in the safe condition), or it might kill the dog (more likely in the danger condition). Thus, “swerve” refers to the percent of dogs neither killed by the car nor actively saved. “Killed” refers to the percent of dogs killed by the car. Note the similarity in proportions of save/swerve/killed across the long (Experiment 1) and short (Experiment 2) versions of the task. This supports the idea that people choose a ‘golden balance’ level of fatigue which they sustain across the whole block. At that level, their cost of effort matches the average disasters averted for each trial. Note also that the higher effort exerted in the danger condition (see Results section 1) only partially mitigated outcomes in the high danger condition: participants suffered about 25% ‘dog deaths’ in high threat vs. 8% in low-threat. This is what one would expect if participants already operated in the low-threat condition under considerable fatigue. As we can expect cost to be proportional both to effort but also to fatigue (and hence, as fatigue increases with effort, to a power of effort greater than one), balancing losses with fatigue will have a reduced effect in moving from low to high threat.

#### Experiment 2

We replicated the analyses in Experiment 1, first testing for normality of the dataset. The Kolmogorov-Smirnov test with Lilliefors correction was again consistent with normally distributed data. A 2-by-2 ANOVA showed no main effect of block number, but replicated the significant effect of disaster condition (*p* < 0.001, η_p_^2^ = 0.249). Once again, participants exerted significantly more effort in the high probability disaster blocks than in the low probability disaster blocks, *t*(89) = 5.43, *p* *<* 0.001. See Table 1 for full statistics, Table 2 for outcomes experienced, and Fig. 3b for overall performance.

### Subjective emotion ratings vary by baseline probability of ‘disaster’

#### Experiment 1

**Figure 4:**
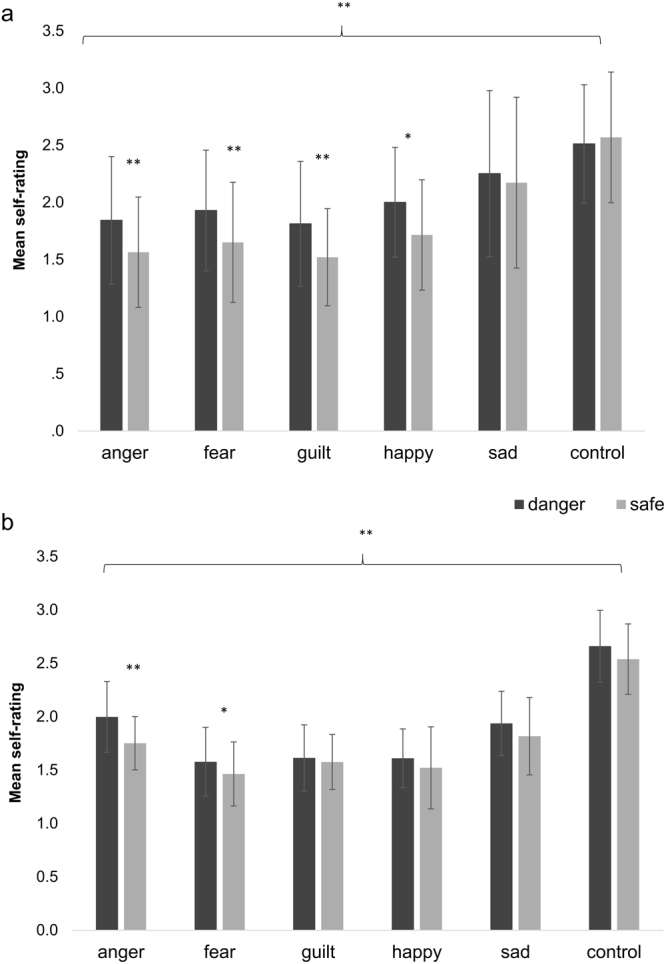
Effect of danger condition on emotion self-rating. (**a**) In Experiment 1, there was a main effect of emotion, block number, and danger condition, with danger condition increasing subjective emotion rating for both negative (*p* < 0.001) and positive (*p* = 0.04) emotions (**b**) In Experiment 2, there was a main effect of emotion and danger condition, with danger condition generally increasing subjective emotion rating for both negative (*p* = 0.003) and positive emotions (*p* = 0.039). *p < 0.05 (does not survive Bonferroni correction for multiple comparisons); **p < 0.001 (survives Bonferroni correction for multiple comparisons). Error bars represent 95% confidence intervals.

In Experiment 1, the result of the KS test with Lilliefors correction for each of the six emotions was consistent with Gaussian data, all *p* > 0.05. Therefore we ran a 2-by-2-by-6 ANOVA (block number, danger condition, and emotion category) finding a main effect of emotion (*p* < 0.001, η_p_^2^ = 0.281), block number (*p* = 0.01), and danger condition (*p* < 0.001, η_p_^2^ = 0.496; Fig. 4a), which manifested as an overall increased emotion ratings in high danger blocks. There was also a significant emotion-by-danger interaction, (*p = *0.001), and no interaction between this and the main effect of block. There was no effect of age on the interaction between emotion and danger condition or gender. See Table 3 for full statistics.

**Table 3:** Full statistics for Experiments 1 and 2 for analyses of the effect of disaster condition on emotions.

	Effect of block number	Effect of emotion type	Effect of danger condition	Emotion-by-danger interaction	Emotion-by-danger-by-block interaction	Effect of age on emotion	Effect of gender on emotion
Exp. 1	F(1,27) = 7.62, p = 0.01	F(5,135) = 10.55, p < 0.001	F(1,27) = 26.53, p < 0.001	F(5,135) = 4.23, p = 0.001	F(5,135) = 1.34, p > 0.250	F(5,65) = 0.6, p > 0.250	F(5,65) = 1.56, p = 0.176
Exp. 2	F(1,89) = 2.10, p = 0.150	F(5,445) = 34.11, p < 0.001	F(1,89) = 9.01, p = 0.003	F(5,445) = 2.72, p = 0.019	F(5,445) = 0.82, p > 0.250	F(5,90) = 0.94, p > 0.250	F(5,90) = 0.96, p > 0.250

We used a planned linear contrast to test our hypothesis that negative emotions would be increased in high probability disaster blocks, collapsing across anger, fear, guilt, and sadness ratings. Our hypothesis was strongly supported, *t*(27) = 6.09, *p* < 0.001, with self-report of negative emotions higher in danger blocks (*M* = 1.96, *SD* = 0.58) than in safe blocks (*M* = 1.73, *SD* = 0.55). It was interesting that a similar contrast collapsing across positive emotions (happiness and feeling in control) individuated an effect in the same direction *t*(27) = 2.16, *p* = 0.04; that is, participants tended to report more positive emotions in danger conditions (*M* = 2.26, *SD* = 0.35) than in safe conditions (*M* = 2.14, *SD* = 0.48). However, the positive emotion contrast was post-hoc and does not survive Bonferroni correction for multiple comparisons: we used a Bonferroni adjusted alpha level of 0.006 per test (0.05/8). Post-hoc linear contrasts revealed that ratings of anger (*t*(27) = 4.54, *p* < 0.001), fear (*t*(27) = 4.32, *p* < 0.001), but also guilt (*t*(27) = 4.10, *p* < 0.001) and happiness (*t*(27) = 3.38, *p* = 0.002) were significantly greater in high-danger blocks. Presumably, guilt emotions were related to trials where the participant failed to save the dog, while happiness ratings were related to successful saves. There was no significant difference in ratings of sadness (*t*(27) = 0.91, *p* > 0.250) or feeling in control (*t*(27) = −0.71, *p* > 0.250).

#### Experiment 2

In Experiment 2, we again failed to reject the hypothesis that the subjective emotion rating data was not normally distributed (KS test with Lilliefors correction for each emotion *p* > 0.05). Therefore we ran a 2-by-2-by-6 ANOVA (block number, danger condition, and emotion category) finding a main effect of emotion (*p* < 0.001, η_p_^2^ = 0.277), no effect of block number, and a main effect of danger condition *(p* = 0.003, η_p_^2^ = 0.092 (Fig. 4b), which again manifested as overall increased emotion ratings in high danger blocks. There was again a significant emotion-by-danger interaction, (*p* = 0.019), and no interaction between this and the main effect of block. There was again no effect of age or gender on the interaction between emotion and danger condition. See Table 3 for full statistics.

A planned linear contrast again confirmed that negative emotions were increased in danger (collapsing across anger, fear, guilt, and sadness ratings) (*M* = 1.78, *SD* = 0.52) relative to safe blocks (*M* = 1.65, *SD* = 0.47), *t*(89) = 3.03, *p* = 0.003. We also replicated our surprising finding that participants reported more positive emotions in danger conditions (*M* = 2.14, *SD* = 0.53) than in safe conditions (*M* = 2.03, *SD* = 0.45), *t*(89) = 2.10, *p* = 0.039, but again, this did not survive Bonferroni correction for multiple comparisons: we used a Bonferroni adjusted alpha level of 0.006 per test (0.05/8). Here, post-hoc linear contrasts revealed that ratings of anger (*p* < 0.001) were greater in the high-danger condition, though fear (*p* = 0.020), sadness (*p* = 0.121), control (*p* = 0.120), happiness (*p* = 0.103) and guilt (*p* > 0.250) did not reach significance.

### The propensity to increase effort in high-danger conditions reflects subjective emotional state

#### Experiment 1

We tested whether negative emotionality reflected behavioral motivation by calculating two variables: (1) the difference in negative emotions in danger blocks minus safe blocks; (2) a ‘defensive effort ratio’ describing the degree to which each participant’s vigor was modulated by danger condition, i.e. [effort in danger blocks/effort in safe blocks]. In Experiment 1, this ratio did not correlate with the difference in negative emotions in danger blocks minus safe blocks (*p* > 0.250). However, sufficient statistical power to detect correlation effect sizes, which are typically small-to-moderate, requires a larger sample size (e.g., a correlation effect size of Cohen’s d = 0.3 requires a sample size of 82 to achieve 80% power).

#### Experiment 2

**Figure 5:**
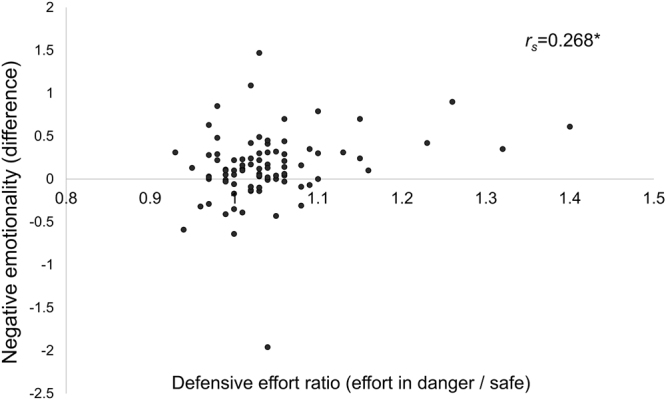
Relationship between defense-related changes in vigor and negative emotionality. A non-parametric correlation test (Spearman’s Rank Order correlation coefficient) revealed a significant relationship between each participant’s defensive effort ratio and the degree to which danger condition increased self-reports of negative emotionality. That is, participants whose negative emotions were most increased in high-danger blocks were also those who modified their behavior most according to probability of disaster. *p = 0.011. Of note, one clear outlier is visible (negative emotionality = −1.8, reporting more negative emotions in the safe blocks than danger blocks), but excluding this participant strengthened the correlation (*p* = 0.002).

With the N = 90 from Experiment 2, we again tested whether a propensity to increase effort in high-danger conditions correlated with subjective emotional state. The distribution of participants’ defensive effort ratios did not conform to assumptions of normality (KS test with Lilliefors correction: D(90) = 0.191, *p* < 0.001). We therefore calculated Spearman’s Rank-Order correlation coefficient to test whether each participant’s defensive effort ratio correlated with the degree to which their negative emotionality was affected by danger condition. We found support for this hypothesis, demonstrating that participants whose negative emotions were most affected by disaster condition were also those who modified their effort most according to probability of disaster, *r*_*s*_ = 0.268, *p* = 0.011 (Fig. 5). There was a clear outlier with respect to negative emotionality (see Fig. 5), but excluding this participant only strengthened the correlation effect, *r*_*s*_ = 0.317, *p* = 0.002.

### The influence of outcomes on emotions and effort

We additionally tested how outcomes experienced (saving the dog, seeing the dog killed, or the car swerving) affected negative emotionality and effort, finding that both were substantially affected by outcome, as well as danger condition (see Supplementary Materials S4).

## Discussion

We show that humans modulate effortful behavior to avoid highly salient negative outcomes (‘catastrophes’) in a pattern qualitatively consistent with predictions from computational models of vigor^11^. It is of scientific and clinical importance that this modulation reflects internal subjective emotions evoked by real or potential losses. Our paradigm has particular relevance for understanding adaptive and maladaptive effortful avoidance. The latter is a key phenotype in several neuropsychiatric disorders^21^. Commonly in OCD, patients develop maladaptive behaviours, or compulsions, as a result of their excessive, intrusive fears of or conflicts about causing harm to others^1–3^. Patients believe that not engaging in these compulsions–counting, checking, washing their hands–might cause injury or death to someone else^3^ (e.g. ‘if I step on a crack, I’ll break my mother’s back’).

These actions are extremely effortful: even in healthy individuals, keeping safe is demanding. It involves balancing the probability of a disaster occurring with the effort necessary to reliably avoid such an occurrence. By varying the probability of disaster we were able to measure how exerting effortful behavior depends on the likelihood of a negative event. In our study human subjects used a baseline probability of disaster to modulate their vigor, akin to findings using monetary incentives which showed a similar relationship between vigor and reward rate^12^. Nonetheless, it would be interesting to compare these findings directly with a monetary incentive version of the task: while monetary rewards have been used in many vigour paradigms before^12, 22, 23^, they have not been directly compared with non-monetary gains or losses such as ours. This would be an important avenue for future research. Crucially, in our case, aversive outcomes had no effect on subjects’ monetary gains, rendering it more likely that our assay relied on subjective mechanisms normally recruited in defensive avoidance. Far from being alternatives to material reinforcers, subjective emotions characterize how a subject mentalizes their evaluation of a situation and their own intentionality within it^24^. The degree to which defensive mechanisms were recruited is, however, likely to be more variable across individuals is the case with real disasters. While some subjects modulate their effort dramatically according to the likelihood of the negative events, others’ behavior changed less, or not at all.

We predicted that negative emotionality would be associated with aversive vigor, which our data supported. We specifically hypothesized that negative emotions as a whole, rather than specific emotions, would relate to effort. This was premised on the idea that verbal descriptions of negative emotion vary between individuals, such that one participant might use “anger” words to describe negative emotionality (due to cultural norms or linguistic style), while another might use “sadness” words. Our data is consistent with this hypothesis: participants whose emotions were most dependent on the state of the task (high- or low-danger) were also those who altered their behaviour most. If they thus sought to avoid experiencing aversive emotions in subsequent trials, this would speak to the inherent human capacity to mentalize one’s own future states. The role of negative emotionality in driving defensive avoidance may be model-based, but not necessarily so. Those more prone to negative emotionality (due to epigenetic influence, for example) may endow effort with greater ‘cached’ value, as per Mowrer’s two-factor theory where this value drives avoidance^25, 26^. More generally, our finding that the propensity to increase effort in high-danger conditions reflects subjective emotional state hints at the importance of emotion in motivation towards effortful behavior. Our supplemental analyses (S4) begin to answer how perception, that is, the outcome participants experience, influences both emotion and effort. However, our results do not speak directly to causality in the relationship between action and emotion. Thus, further research on this topic is needed to clarify the complex relationship between perception, emotion, and action.

Regarding neural implementation, the cardinal neurotransmitter studied in appetitive vigor has been dopamine^14–16^. Theory predicted that dopamine signals an integrated reward rate, including opportunity cost^10^. This prediction was supported in a finding where boosting dopamine using its precursor levodopa increases the influence of the average reward on effort^17^. However, this effect has only been tested in the reward domain and it is hypothesized that serotonin plays a greater role in determining vigor in the context of punishment^17^. This suggestion is supported by its effect on response inhibition in the context of punishment^27^. In our task, reinforcement-learning theory predicts that serotonin may report the rate of realized aversive outcomes while dopamine may report the rate of successfully avoided outcomes^11^. Genetic polymorphisms, epigenetic, and pharmacological factors affecting serotonin transmission should thus have a commensurate effect on aversive vigor, and, by extension, avoidance behavior.

The design of our study, while it endeavored to be as immersive as possible, was subject to important individual differences. Thus, some participants worked much harder to save the virtual dog than others, making the paradigm an imperfect representation of universally-avoided disasters in the world. However, this limitation may also represent a potential strength: the propensity to engage effortfully with a task involving imaginary consequences might reflect real-world efforts to avoid highly unlikely, imagined disasters (such as those in paranoid thoughts, for example). A second limitation of our task is its fixed block order. We did use a break halfway through to limit confounding by increased fatigue. We incorporated this rest so that the second low-traffic block was completed after substantial recovery: if fatigue accounted for our key findings, we would expect this low-threat ‘recovery block’ to resemble the first, high-threat block. Nonetheless, it is important to randomize block order in future research to verify these findings.

Our findings suggest that avoidance behavior is coupled with subjective negative emotion, lending way to the possibility that aberrant negative emotions such as anger and fear could play a key role in driving pathological avoidance. Pathological avoidance behavior is often driven by a fear of causing harm to others, a symptom seen in patients with OCD and some psychotic disorders, and which our paradigm went some way towards evoking. Our paradigm may also be relevant for disorders of excessive avoidance of disaster-to-self, to the degree that other-evaluation and self-evaluation have been found to share overlapping mechanisms^28^. Thus our task has the potential to characterize emotions and behaviors common across several psychopathological and neurological conditions. Future research should employ even more refined paradigms in clinical populations, combined with computational modelling, to understand the mechanisms of avoidance behaviour from a transdiagnostic point of view.

### Data availability

All data and stimulus materials are freely and publicly available via the Open Science Framework. See: Nord, C. (2016, August 2). Vigour and catastrophe. osf.io/kqw6r.

## Electronic supplementary material

Vigour in active avoidance
