# Supplementary material for: Vigour in active avoidance
Source: Sci Rep. 2017 Mar 3;7:60. doi: 10.1038/s41598-017-00127-6 (PMC5427871; doi:10.1038/s41598-017-00127-6)
Supplement: Supplementary file 1 — Vigour in active avoidance [file 41598_2017_127_MOESM1_ESM.pdf]

Supplementary Materials

Vigour in active avoidance

Camilla L Nord[1*], Gita Prabhu[2], Tobias Nolte[2,3], Peter Fonagy[3,5], Ray Dolan[2,4], Michael Moutoussis[2]

[1]Institute of Cognitive Neuroscience, University College London, London, UK

[2]Wellcome Trust Centre for Neuroimaging, UCL, 12 Queen Square, London, UK

[3]Anna Freud Centre, London, UK

[4]Max Plank UCL Centre for Computational Psychiatry and Ageing Research, London, UK

[5]Research Department of Clinical, Educational, and Health Psychology, University College London, London, UK

**Please address correspondence to:*

Camilla L Nord
Institute of Cognitive Neuroscience
17 Queen Square, London, U.K.
WC1N 3AZ
camilla.nord.11@ucl.ac.uk

S1. Selected responses from immersion task:

Participant 1: *"I would be quite distraught, all the memories I had shared with my dog would come back to me. A great sense of loss would overwhelm me. If I knew I could have prevented her death, I would everything in my power to make it happen. My dog is my pet, my friend, my companion for all these years."*

Participant 2: *"I would feel like a member of the family has been lost, as cliché as it sounds. Pets make for good friends really and their loss would be very much regretted. I would have felt very relieved and happy if I could have prevented it".*

Participant 3: "*When it happened, I was overcome with a sense of loss, as if a long-term friend had died. It was a real sense of loss and I found my emotions difficult to control".*

S2. Emotion word selection task

Five sequential emotional word choices were done on the computer, and each choice was preceded with the question, referring to the written immersion task:

“Which word describes best how you felt at any point during the task?”

Each participant selected one word from each category, and these word selections were automatically input into the in-task self-rating questions. In addition to these five categories, all participants were automatically asked the question: “To what degree did you feel out of control when trying to save the dog?”.

For the five sequential categories (in this order), the following words were offered as options:

Fear words	Anger words	Guilt words	Sad words	Happy words
“Afraid”	*“Angry”*	*“Guilty”*	*“Sad”*	*“Happy”*
“Scared”	*“Hostile”*	*“Ashamed”*	*“Blue”*	*“Joyful”*
“Frightened”	*“Irritable”*	*“Blameworthy”*	*“Downhearted”*	*“Delighted”*
“Nervous”	*“Scornful”*	*“Angry at self”*	*“Alone”*	*“Cheerful”*
“Jittery”	*“Disgusted”*	*“Disgusted”*	*“Lonely”*	*“Excited”*
“Shaky”	*“Loathing”*	*“Dissatisfied”*		*“Enthusiastic”*
				“Lively”
				“Energetic”

S3. Representative example of effort in trial

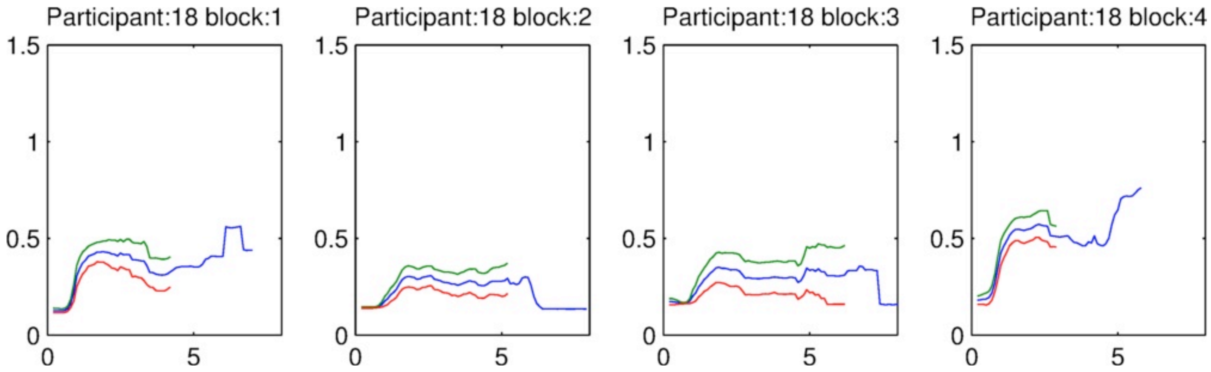

Figure S3. Representative example of time dependence of effort within trial. The y axis indicates the percentage of maximum effort exerted versus time in seconds (x axis). The blue line indicates average effort. Green and red lines show the confidence interval when at least 9 trials were available at that duration. Note, if there were no effort-onset effect as per (Dayan, 2012), near-maximal effort would be established with negligible latency. The participant instead makes sure that their chosen plateau is reached at about 1-2 sec, well before the maximum probability of the aversive event occurring (at 5 sec).

S4. Emotion and effort – outcome analysis.

We conducted a supplementary analysis of the effect of outcome on negative emotionality across both experiments combined. We conducted a 3-by-2 ANOVA examining the effect of outcome (the dog being saved, the car swerving, or the dog being killed), danger condition on subjective negative emotions reported. Because not all participants experienced emotion questions after every outcome, in each danger condition, we combined data from the two experiments. We found a very large effect of outcome on negative emotions, with the most negative emotions reported following a killed dog, and the lowest negative emotions following the dog being saved ($F(2,90)=26.885, p<0.001, \eta_p^2=0.374$). There was also a substantial effect of danger $F(1,45)=19.123$, $p<0.001$, $\eta_p^2=0.298$, with negative emotions generally higher in danger blocks, and an interaction between outcome and danger $F(2,90)=3.318$, $p=0.015$, $\eta_p^2=0.089$). See Figure S2.

We conducted a 3-by-2 ANOVA examining the effect of outcome (the dog being saved, the car swerving, or the dog being killed) and danger condition on subsequent effort. We combined data from the two experiments: we found a very large effect of outcome on effort, with the most effort in both conditions occurring after a saved dog ($F(2,96)=23.06, p<0.001, \eta_p^2=0.13$). Again, there was a substantial effect of danger ($F(1,48)=7.228, p=0.01$), with effort consistently higher in danger blocks across all outcomes. There was no interaction between outcome and danger condition ($F(2,96)=0.11, p=0.896$).

We also explored the relationship between outcome, action, and emotion further by calculating, for each participant, whether their negative emotions reported following each outcome exceeded their average negative emotions

reported. Using a Chi-square test, we found that people report more than their average negative emotion directly after the dog is killed, rather than the saved or swerved outcomes ($X^2(2)=17.765$, $p<0.001$ for the danger condition; $X^2(2)=11.178$, $p=0.004$ for the safe condition). We then tested whether experiencing more-than-average negative emotion was associated with performing more-than-average effort. In a second Chi-square test, we found that emotions were not associated with subsequent effort in the safe blocks $X^2=0.010$, or danger blocks, $X^2=0.251$, both $p>0.250$.

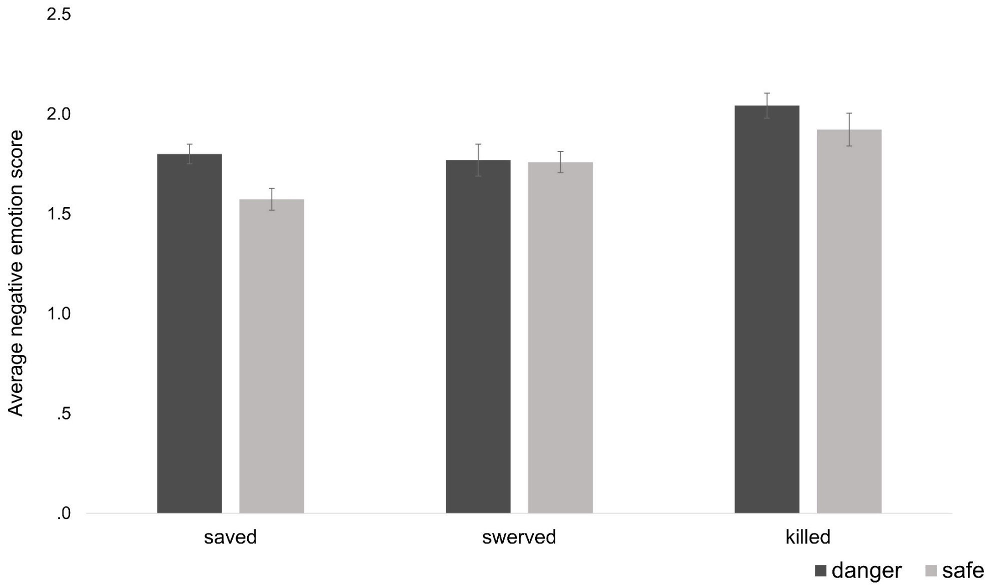

Figure S4. Effect of outcome on participants' subsequent rating of negative emotions.
